# Skeletal intramyocellular lipid metabolism and insulin resistance

**DOI:** 10.1007/s41048-015-0013-0

**Published:** 2015-10-23

**Authors:** Yiran Li, Shimeng Xu, Xuelin Zhang, Zongchun Yi, Simon Cichello

**Affiliations:** Department of Biological Science and Biotechnology, School of Biological Science and Medical Engineering, Beihang University, Beijing, 100191 China; National Laboratory of Biomacromolecules, Institute of Biophysics, Chinese Academy of Sciences, Beijing, 100101 China; University of Chinese Academy of Sciences, Beijing, 100049 China; Capital University of Physical Education and Sport, Beijing, 100191 China; School of Life Sciences, La Trobe University, Melbourne, VIC 3086 Australia

**Keywords:** Skeletal muscle, Intramyocellular lipid, Insulin resistance, Free fatty acid

## Abstract

Lipids stored in skeletal muscle cells are known as intramyocellular lipid (IMCL). Disorders involving IMCL and its causative factor, circulatory free fatty acids (FFAs), induce a toxic state and ultimately result in insulin resistance (IR) in muscle tissue. On the other hand, intramuscular triglyceride (IMTG), the most abundant component of IMCL and an essential energy source for active skeletal muscle, is different from other IMCLs, as it is stored in lipid droplets and plays a pivotal role in skeletal muscle energy homeostasis. This review discusses the association of FFA-induced ectopic lipid accumulation and IR, with specific emphasis on the relationship between IMCL/IMTG metabolism and IR.

## INTRODUCTION

Insulin resistance (IR) is defined as the inability of target tissues to increase glucose uptake in response to insulin, which eventually leads to type II diabetes mellitus (T2DM) (Eckel et al. 2005; Samuel and Shulman 2012). IR occurs in virtually all patients with T2DM, most of whom are obese and have fat maldistribution. In addition to the association between T2DM and generalized obesity, many studies have also revealed associations between IR and body fat distribution, particularly the fat distribution in skeletal muscle, as this tissue is responsible for the majority of whole-body insulin-stimulated glucose disposal. Skeletal muscle insulin resistance is central to the pathogenesis of T2DM (Björnholm and Zierath 2005). Human skeletal muscle is a heterogeneous organ consisting of two phenotypically distinct kinds of muscle fibers. The histochemical staining for pH-sensitive myosin ATPase activity reveals two major classes of fiber types, namely type I and type II fibers. Type I (slow twitch) muscle fibers tend to be oxidative, whereas type II (fast twitch) fibers are glycolytic (Raben et al. 1998; Pette et al. 1999). Both type I and type II muscle fibers are insulin sensitive (James et al. 1985; Kern et al. 1990).

All types of lipids within myocytes are referred to as intramyocellular lipids (IMCLs), which are composed chiefly of triacylglycerol (TAG) but also include diacylglycerol (DAG), sphingolipid, and phospholipid. The accumulation of IMCL is essential for metabolism and physical exercise. Recently, the excess accumulation of IMCL has been linked to regional fat distribution, gaining considerable attention because of its association with IR. Paradoxically, both trained athletes and T2DM patients possess higher IMCL than normal healthy individuals. However, only athletes possess a high oxidative capacity in muscle and thus enhanced insulin sensitivity. This phenomenon is called the “athlete’s paradox”. One type of IMCL that is stored mainly in lipid droplets (LDs), namely intramuscular triglyceride (IMTG), plays an important role in maintaining lipid homeostasis, including lipid metabolism, membrane trafficking, cell signaling, hormone production, and other molecular events. The disorder of IMCL and its derivatives leads to many metabolic diseases.

## GLUCOSE-FATTY ACID CYCLE (RANDLE CYCLE)

In the early 20th century, it was recognized that both fat and carbohydrate are used as fuel during physical exercise (Krogh and Lindhard 1920). In 1926, Himwich and Rose examined muscular fuel utilization by measuring arteriovenous differences in oxygen and carbon dioxide across skeletal muscle in dogs during rest and exercise, and in fed and fasted states. The results showed that the respiratory quotient of the exercising muscle was not unity, which indicated that not only carbohydrates but also non-carbohydrates were used in muscular exercise (Himwich and Rose 1926). Later, Fritz et al. observed that fatty acid was oxidized in skeletal muscle during both rest and activity. This provided evidence that muscle oxidizes lipid to support muscle contraction (Fritz et al. 1958). Of particular note is the 1963 *Lancet* publication by Randle et al., which proposed a “glucose-fatty acid cycle”, also known as the Randle cycle (Randle et al. 1963). The Randle cycle described the fuel flux between tissues as well as fuel selection by tissues. The original biochemical mechanism proposed that glucose oxidation was inhibited by fatty acids. Subsequently, lipid metabolism and glucose metabolism were linked, and researches accumulated in this field. Soon thereafter, researchers observed that IMTG could be used as fuel during exercise, and IMTG accumulation was found to be associated with IR in various studies (Watt 2009).

The Randle cycle has been contested as ignorant, because it postulates an exact correlation of metabolic fuel with competition between glucose and fatty acid during their oxidation by muscle and adipose tissue (Randle et al. 1963). Because of the prevalence of obesity and T2DM, researchers have paid increasing attention to this field. Recently, the understanding of the relationship between lipid metabolism (e.g., IMCL) and glucose metabolism (e.g., especially its related disorder, IR) has been intensely developed.

## THE EFFECTS OF DIFFERENT TYPE OF LIPIDS ON INSULIN RESISTANCE

### Triacylglycerol

Intramuscular lipids are stored predominantly as IMTG within LDs. The presence of IMTG was first described by Denton and Randle in 1967 (Denton and Randle 1967) and corroborated by Van Loon in 2004 (Van Loon 2004). The study by Van Loon used stable isotope methodology, ^1^H-magnetic resonance spectroscopy, and electron and/or immunofluorescence microscopy to confirm that IMTG functions as an important substrate source during exercise. This study also found that up to 60%–70% of IMTG can be depleted in type I muscle fibers during prolonged moderate intensity exercise in trained individuals; this oxidation accounts for up to 50% of total lipid oxidized as a fuel source in the exercising muscle (Van Loon 2004). The application of these analytical techniques has facilitated the examination of IMTG and made it possible to study the function of IMTG as a metabolic fuel and its relationship with IR. Using ^1^H-magnetic resonance spectroscopy, researchers demonstrated that IMTG could be used as a fuel source by exercising muscle (White et al. 2003) and depleted in both acute and long-term forms of exercise. Again, with the concept of the athlete’s paradox, the concentration of IMTG is adaptively increased in endurance-trained individuals and in response to exercise training interventions, which paradoxically does not adversely affect insulin sensitivity and oxidative capacity (Goodpaster et al. 2001; Russell et al. 2003). In contrast, higher IMTG content is also observed in obese and T2DM individuals, or others whose insulin-sensing capability is impaired. Human studies have proposed the hypothesis that IMTG accumulation is associated with IR (Pan et al. 1995). This hypothesis has been refined over the last 15 years with more experimental and statistical results supporting the theory that accumulation of IMTG has important contributions to the development of skeletal muscle IR (Jacob et al. 1998; Bachmann et al. 2001; Goodpaster et al. 2001; Jimenez-Caballero et al. 2008; Anastasiou et al. 2009). For example, in a study of 19 non-diabetic obese and 11 diabetic obese individuals, Anasious and colleagues observed higher levels of IMTG in the diabetic obese group compared with the non-diabetic obese group, and that DAG levels were not significantly different between the study groups (Anastasiou et al. 2009). Another study from Schenk and colleagues observed that the prevention of fatty acid-induced IR following acute exercise was accompanied by enhanced skeletal muscle protein expression of key lipogenic enzymes, and further increased the rate of muscle TAG synthesis in humans (Schenk and Horowitz 2007). Furthermore, TAG-induced IR may be muscle type dependent. As previously mentioned, most IMTG is contained and used within type I muscle fibers, as IMTG content is threefold higher in type I oxidative fibers than type II glycolytic fibers (Shaw et al. 2012). This suggests that type I muscle fibers are more important in lipid toxicity (Coen and Goodpaster 2012) and abnormal lipid metabolism-induced IR. Moreover, human skeletal muscle IR is related to excess IMTG content in type I but not type II myocytes, greater ceramide content, and alterations in gene expression associated with lipid metabolism (Coen et al. 2010). Interestingly, the overexpression of acyl-CoA:diacylglycerol acyltransferase 2 (DGAT2) in type II glycolytic muscle of mice increases TAG, ceramide, and unsaturated long chain fatty acyl-CoA (LCFA-CoA) in skeletal muscle content of young adult mice, which is accompanied by impaired insulin signaling and insulin-mediated glucose uptake in glycolytic muscle and further impaired whole-body glucose and insulin tolerance (Levin et al. 2007). Moreover, diacylglycerol acyltransferase 1 (DGAT1)-deficient mice are resistant to diet-induced obesity through a mechanism involving increased energy expenditure. Chen and colleagues showed that these DGAT1-deficient mice have decreased levels of skeletal muscle TAG after induction of high-fat-diet-induced obesity, in addition to increased sensitivity to insulin and leptin (Chen et al. 2002). Their findings also demonstrated that DGAT1 deficiency in mice enhances insulin signaling in the skeletal muscle and white adipose tissue (WAT), in part through altered expression of adipocyte-derived factors that modulate insulin signaling in peripheral tissues (Chen et al. 2004). The results of these animal studies suggest that IMTG accumulation may be a causative factor of IR, a fact that is now widely accepted (Saltiel 2000).

**Fig. 1:**
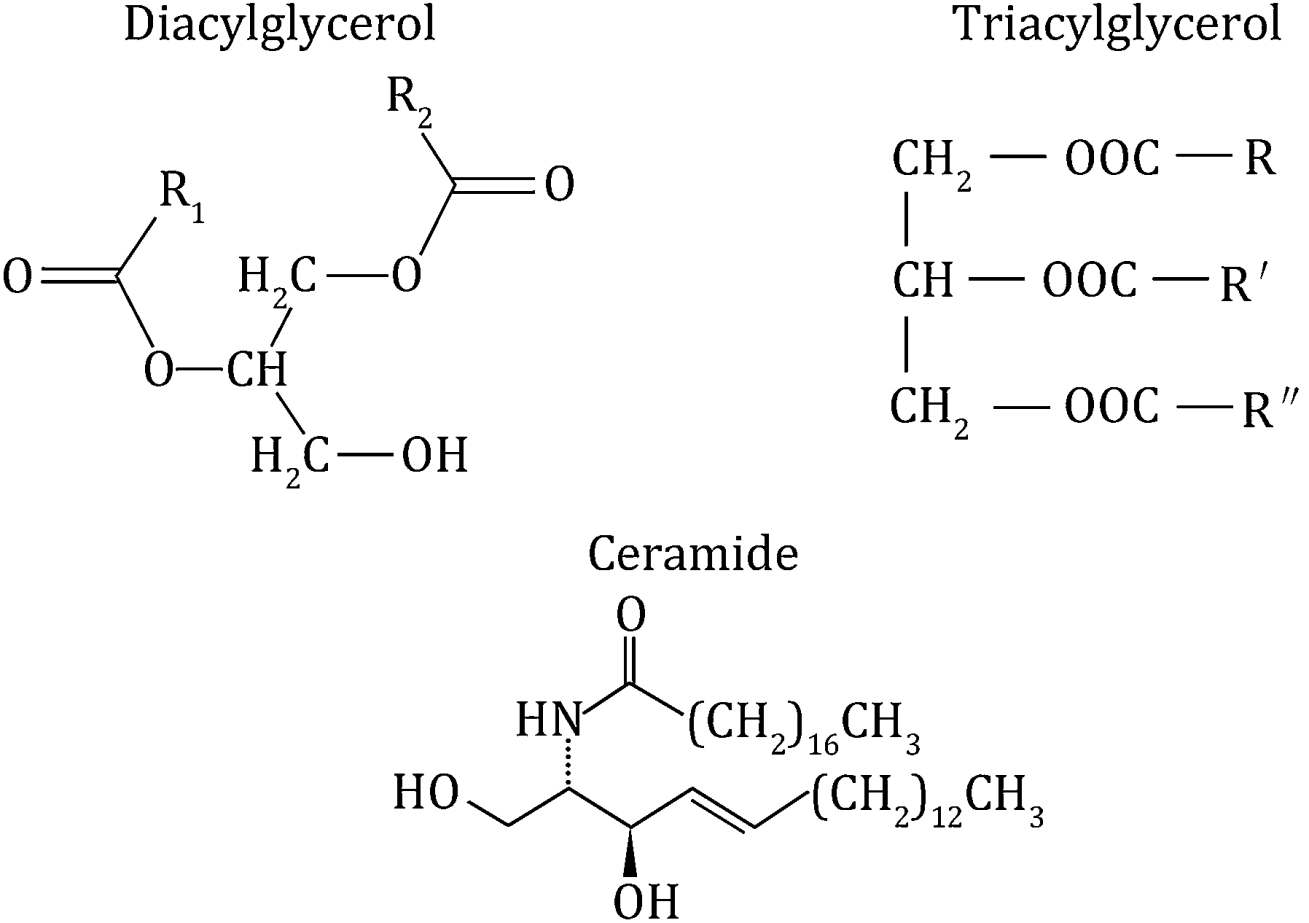
Chemical structures of diacylglycerol, triacylglycerol, and ceramide. R1 and R2 in diacylglycerol, and R, R′, and R″ in triacylglycerol represent an alkyl or an alkenyl hydrocarbon chain of a fatty acid that is esterified on the glycerol, respectively

Despite the evidence pointing to the role of IMTG in IR, it is difficult to propose that IMTG alone causes IR within the skeletal muscle tissue for several reasons. Elevation of IMTG content in T2DM is usually accompanied by higher concentrations of lipotoxic intermediates such as DAG and ceramide. Both of these metabolites inhibit insulin signaling and interfere with insulin-stimulated glucose metabolism (Samuel and Shulman 2012), and thus it is difficult to propose that IR is induced mainly by IMTG or other lipids. Furthermore, the existence of the athlete’s paradox causes skepticism and prevents total acceptance of the hypothesis that IMTG causes IR within skeletal muscle. Finally, overexpression of DGAT1 in mouse skeletal muscle rescues high-fat-diet-induced IR, accompanied by high TAG levels in skeletal muscle (Liu et al. 2007) (Fig. 1).

Although many studies have shown the tight connection between IMTG and IR, the mechanism linking IMTG and IR needs to be further investigated. It seems that the types of muscle fibers and lipid metabolites, such as DAG and ceramide, play important roles in the relationship between accumulation of IMTG and IR.

### Diacylglycerol

It is widely accepted that sn-1,2-Diacylglycerol (DAG) derived from phospholipids by phospholipase C is an important lipotoxic mediator in IR development, although the specific mechanism remains elusive. Several clinical studies demonstrated that compared with lean controls, the intramyocellular DAG content of the vastus lateralis muscle is elevated in obese and T2DM patients (Moro et al. 2009; Bergman et al. 2012) and is also increased following acute IR induced by lipid infusion (Itani et al. 2002). Increasing DAG accumulation in skeletal muscle by altering expression of adipose triglyceride lipase (ATGL) and hormone-sensitive lipase (HSL) leads to the disruption of insulin signaling, and contributes to IR in humans (Badin et al. 2012). The relationship between DAG accumulation and IR is further confirmed in a study showing that reduced activity of diacylglycerol kinase-δ leads to high intramuscular DAG content in individuals with poorly controlled T2D (Chibalin et al. 2008). Moreover, after several weeks of endurance training and weight loss, skeletal muscle DAG content is decreased, accompanied by a parallel improvement in insulin sensitivity (Dube et al. 2011). In conclusion, these results support the theory that DAG accumulation is associated with IR.

Mechanistically, DAG is a second messenger that activates members of the protein kinase C (PKC) family. The PKC family is divided into three isoforms: classical PKC (α, βI, βII, γ), novel PKC (δ, ε, η, θ), and atypical PKC (ζ, λ). Once activated, PKCs phosphorylate serine residues on insulin receptor substrate 1 (IRS-1), inhibiting the kinase activity and subsequently reducing activation of PI3-kinase and PKB/Akt (Timmers et al. 2008). As a result, insulin-stimulated GLUT4 translocation to the plasma membrane is impaired, therefore IR occurs. Furthermore, it is commonly accepted that in individuals with obesity and T2DM, the elevated DAG content might also increase the activity of PKCs. Itani and colleagues observed that lipid-induced IR in human muscle infused with lipids and insulin over a 6-h time period is associated with changes in DAG, PKC, and in IkB, the downstream signaling molecule of NF-κB (Itani et al. 2002). Furthermore, some researchers hypothesize that DAG mediates IR mainly through novel PKCs (Erion and Shulman 2010). PKCθ is a crucial component in skeletal muscle, and is also the most abundantly expressed PKC in this tissue. Knockout of PKCθ in skeletal muscle prevents fat-induced defects in insulin signaling and glucose transport (Kim et al. 2004). In addition, both transgenic mice with muscle-specific expression of dominant negative *PKCθ* and *PKCθ*-knockout mice exhibit age-associated or diet-associated obesity and whole-body IR (Serra et al. 2003; Gao et al. 2007). When combined, these results support the hypothesis that the accumulation of DAG in skeletal muscle leads to the activation of novel PKCs and ultimately results in IR.

On the other hand, others have shown dissociation between DAG accumulation and IR. Some studies have reported that DAG content in skeletal muscle is not elevated during obesity (Anastasiou et al. 2009), with IR (Hees et al. 2001), or in obese IR (Coen et al. 2010) compared with insulin-sensitive obese subjects. In addition, in highly trained athletes, total myocellular DAG is markedly higher, corresponding with higher insulin sensitivity (Amati et al. 2010). For animal models, overexpression of the DGAT1 enzyme in muscle results in DAG accumulation and release of IR that was induced by a high-fat diet (Timmers et al. 2010).

Researchers are still struggling to elucidate the relationship between DAG and IR in human muscle and answer the question of why DAG accumulation leads to IR. There are several possible explanations, stemming from different viewpoints, as to why DAG accumulation leads to IR. One possible explanation is the degree of FA saturation in DAG. It has been demonstrated that athletes have a lower degree of DAG saturation compared with sedentary controls (Bergman et al. 2012). Moreover, it has been observed that a higher degree of DAG saturation is associated with IR in men with metabolic syndrome (Hees et al. 2001). Conversely, others have not shown such associations (Coen et al. 2010) or even an inverse relationship (Amati et al. 2010). The subcellular location of DAG accumulation might be another possible factor that could affect the relationship of DAG and IR. DAG is present in the sarcolemma membrane, sarcoplasmic reticulum, LDs, and mitochondrial membrane. The majority of human studies only examine whole-muscle DAG concentration, which could certainly obscure the relationship between subcellular DAG concentration and IR. Moreover, it has been recently shown that membrane DAG is associated with PKC activation and insulin sensitivity in obese T2D subjects and lean athletes (Bergman et al. 2012). Finally, and perhaps most importantly, there are two distinct DAG stereoisomers, 1,3-DAG and 1,2-DAG, which may influence muscle IR to different degrees. Only 1,2-DAG has been associated with insulin signaling (Turinsky et al. 1990), and only the 1,2-DAG stereoisomer can activate PKC; 1,3-DAG lacks this ability (Boni and Rando 1985). It has been suggested that neither ATGL nor HSL has the ability to generate the 1,2-DAG stereoisomer (Zechner et al. 2012); however, this hypothesis presently lacks supporting evidence.

### Ceramide

Ceramide, which belongs to the sphingolipid family, plays a role as an inert structural component of biological membranes. It also acts as an intracellular messenger in various biological mechanisms and as a lipid intermediate widely believed to be the true lipotoxic culprit behind the reported associations between IMTG and IR. In skeletal muscle, ceramide accumulation is associated with a number of cellular stresses, such as reactive oxygen species (ROS) accumulation, inflammation, hypoxia, and as part of a highly conserved stress response. All of these stresses have been identified as key mediators of IR via inhibition of the serine/threonine-specific protein kinase Akt/protein kinase B (PKB) (Chavez et al. 2003; Holland et al. 2012), and as important pathways linking insulin signaling to the translocation of GLUT4 to the sarcolemma, potentially via protein phosphatase 2 (PP2)- and protein kinase C zeta (PKCz)-dependent pathways (Stratford et al. 2004). Ceramide is also linked to mitochondrial dysfunction (Smith et al. 2013), which in turn is implicated in IMCL accumulation and IR (Coen and Goodpaster 2012).

Plasma ceramide targets skeletal muscle in T2DM (Kirwan 2013), and ceramide content is increased in skeletal muscle in obese and insulin-resistant humans. By using euglycemic-hyperinsulinemic clamps with muscle biopsies, it has been observed that muscle ceramide content is significantly correlated with the plasma FFA concentration in lean insulin-sensitive and obese insulin-resistant subjects (Adams et al. 2004). Furthermore, as previously mentioned, human skeletal muscle IR is related to greater IMTG content in type I but not type II myocytes, and it is also related to greater ceramide content, especially in type I myocytes. However, the concentration of DAG is similar in both type I and type II myocytes (Coen et al. 2010).

Similar results have been observed using the in vitro cultivation of human primary myoblast cells. By treating human vastus lateralis muscle with different kinds of FFA, Laura and colleagues observed that the application of palmitate produces more DAG and ceramide in myoblasts in addition to the induction of IR. Furthermore, oleate treatment resulted in an increase in TAG in normal insulin-sensitive muscles. These myoblasts developed IR when treated with cell-permeable analogs of ceramide, and showed normal insulin sensitivity with co-treatment of palmitate and inhibitors of de novo ceramide synthesis (Pickersgill et al. 2007). Coincidentally, inhibition of de novo ceramide synthesis reversed diet-induced IR and enhanced whole-body oxygen consumption (Ussher et al. 2010).

Several hypotheses have been proposed to explain the mechanism by which an increase in ceramide leads to IR. Increased mitochondrial oxidative stress and mitochondrial dysfunction are accepted as important causative factors for IR and T2DM (Kelley et al. 2002; Schrauwen and Hesselink 2004; Lowell and Shulman 2005; Fridlyand and Philipson 2006; Schenk and Horowitz 2007; Fleischman et al. 2009; Hernandez-Alvarez et al. 2010; Meex et al. 2010; Schrauwen et al. 2010; Chow et al. 2012;). The results of a study by Larysa et al. support this idea, as they demonstrated that de novo synthesis of ceramide is involved in palmitate-induced mtROS generation, mitochondrial dysfunction, and insulin signaling (Yuzefovych et al. 2010).

The effects of exercise and training on ceramide metabolism in human skeletal muscle have been previously studied (Helge et al. 2004). Thrush et al. observed an interesting phenomenon; although the inhibition of ceramide accumulation can prevent the detrimental effects of palmitate incubation, a single prior bout of exercise appears to protect the muscle against palmitate-induced IR, which may be independent of the variable ceramide concentration (Thrush et al. 2010). Further research has been performed by Skovbro et al. showing that human skeletal muscle ceramide content is not a major factor in muscle insulin sensitivity (Skovbro et al. 2008). In conclusion, the exact mechanism by which ceramide induces IR is still unclear, but it appears that this relationship might be influenced by skeletal muscle lipid composition.

## EFFECTS OF LIPID COMPOSITION

As mentioned previously, DAG stereoisomers have different effects on skeletal muscle IR. The stereo structures, degree of fatty acid saturation, and the length of the fatty acid chains are all contributing factors to these effects. Oleate (18:1) and palmitate (16:0) are widely used in studies of DAGs and IR, since they are both prevalent plasma FFAs. Many studies suggest that saturated palmitate, but not monounsaturated oleate, induces inter-myocellular IR. In C2C12 myotubes, palmitate, but not oleate, inhibits insulin-stimulated glycogen synthesis, as well as the activation of Akt/PKB, an obligate intermediate in the regulation of anabolic metabolism. Palmitate also induces the accrual of ceramide and DAG, which have been confirmed to inhibit insulin signaling in cultured cells and to accumulate in IR tissues (Chavez and Summers 2003). Moreover, oleate protects rat skeletal muscle cell lines against palmitate-induced IR (Coll et al. 2008; Gao et al. 2009) and blocks palmitate-induced abnormal lipid distribution, endoplasmic reticulum expansion, and stress (Dimopoulos et al. 2006; Peng et al. 2012). These results have also been observed in other cell models (Listenberger et al. 2003). Furthermore, excluding monounsaturated fatty acid, the treatment of these cells with linoleate (C18:2) or n-6 polyunsaturated fatty acid does not alter DAG levels, ceramide levels, or glucose uptake, but increases myotube TAG levels compared with controls that lack the addition of fatty acids (Lee et al. 2006). These studies suggest that saturated fatty acid-induced IR occurs by a mechanism distinct from that of unsaturated fatty acids, and thus is related to the degree of saturation. Furthermore, this mechanism involves elevation of ceramide, which leads to PKB inhibition without affecting IRS-1 function (Schmitz-Peiffer et al. 1999). Unsaturated fatty acid serves as a protective functional factor through the promotion of TAG accumulation, and thereby decreases DAG and ceramide content (Listenberger et al. 2003). There are also other proposed mechanisms, including one involving mitochondria. It has been proposed that mtROS generation is the initial event in the induction of mitochondrial dysfunction and consequently apoptosis, and the inhibition of insulin signaling. The palmitate-induced mitochondrial dysfunction is ameliorated by oleate, which contributes to the prevention of palmitate-induced IR (Yuzefovych et al. 2010). Thus, it appears that the fatty acid concentration is not the only determinant in the induction of IR.

An in vivo study also suggested that dietary fat composition, rather than fatty acid over-supply, is the major determinant of fat-induced IR (Storlien et al. 1991, 2002). Human dietary fat consists of a range of fats including saturated, monounsaturated, polyunsaturated, and trans-unsaturated fatty acids. Previous studies found that high dietary intake of the monounsaturated fatty acid oleic acid, which is abundant in olive oil, is associated with improved insulin sensitivity in the general population, whereas saturated fatty acids (i.e., palmitate) show the opposite effect (Marshall et al. 1997; Soriguer et al. 2004). Furthermore, most animal and cell studies indicate that saturated fatty acids significantly increase IR, whereas n-3 polyunsaturated fatty acids prevent it (Storlien et al. 2002). Animals fed a diet high in n-6 polyunsaturated fat retained insulin sensitivity despite small increases in muscle DAG, which appears to be in a range somewhere between the effects of saturated and n-3 fatty acids (Storlien et al. 1991).

The most popular hypothesis of the complicated phenomenon of IR is that unsaturated FFA helps with the incorporation of saturated FFA into IMTG, accompanied by much less harmful lipid derivatives (e.g., DAG and ceramide) and therefore, muscle cells are protected from IR. Animal feeding or perfusion studies, as well as cell culture studies, have linked saturated fatty acid intake with elevated concentrations of specific lipid messengers in muscle (Lee et al. 2006; Coll et al. 2008; Peng et al. 2012; Sawada et al. 2012; Salvado et al. 2013). Another hypothesis is that the cell membrane fatty acid composition in turn affects cell membrane fluidity and rigidity. In humans and other species, the body is particularly efficient at regulating the components of cell membranes, such as the sarcolemma, which can be influenced by plasma FFA (Storlien et al. 1998). Since the efficiency of signal transduction is highly dependent on the orientation and position of various proteins within the membrane, the fatty acid composition of cellular membranes may play a pivotal role in adequate insulin sensitivity (Corcoran et al. 2007). Furthermore, there are also studies in humans suggesting that the fatty acid composition of phospholipids in the sarcolemma modulates insulin sensitivity (Storlien et al. 1991; Borkman et al. 1993). Animal studies seem to directly demonstrate that saturated FFA-containing membranes promote IR, whereas a high degree of unsaturation in the FFAs in the membranes protects against IR; this has also been noted in humans (Storlien et al. 1996).

## CONCLUSION

Lipid and glucose metabolism are both important for human health, and skeletal muscle is the major organ responsible for balancing the use of lipid and glucose as fuel during complete rest and low-intensity activity. Research has considerably advanced our understanding of intramuscular lipid metabolism and its significance in human health. Although we have been aware of and gained considerable insight into the role of IMCL in metabolism and the development of IR, there are still complex questions to be answered. For now, it seems that maintaining the dynamic lipid balance may be one of the most important functions for human health. In the future, a better understanding of IMCL and IR will serve as a means to cure metabolic diseases and promote human health. Advanced lipidomics, combined with more specific and efficacious small molecule inhibitors, will accelerate research progress toward understanding how specific lipid metabolites influence metabolic homeostasis and finding applicable therapies for IR.
